# Promotion of compound K production in *Saccharomyces cerevisiae* by glycerol

**DOI:** 10.1186/s12934-020-01306-3

**Published:** 2020-02-19

**Authors:** Weihua Nan, Fanglong Zhao, Chuanbo Zhang, Haiyan Ju, Wenyu Lu

**Affiliations:** 1 School of Chemical Engineering and Technology, Tianjin University, Tianjin, 300350 People’s Republic of China; 2 Key Laboratory of System Bioengineering (Tianjin University), Ministry of Education, Tianjin, 300350 People’s Republic of China; 3 SynBio Research Platform, Collaborative Innovation Center of Chemical Science and Engineering (Tianjin), Tianjin, 300350 People’s Republic of China

**Keywords:** Compound K, *Saccharomyces cerevisiae*, Glycerol, Metabolic engineering

## Abstract

**Background:**

Ginsenoside compound K (CK), one of the primary active metabolites of protopanaxadiol-type ginsenosides, is produced by the intestinal flora that degrade ginseng saponins and exhibits diverse biological properties such as anticancer, anti-inflammatory, and anti-allergic properties. However, it is less abundant in plants. Therefore, enabling its commercialization by construction of a *Saccharomyces cerevisiae* cell factory is of considerable significance.

**Results:**

We induced overexpression of *PGM2*, *UGP1*, and *UGT1* genes in WLT-MVA5, and obtained a strain that produces ginsenoside CK. The production of CK at 96 h was 263.94 ± 2.36 mg/L, and the conversion rate from protopanaxadiol (PPD) to ginsenoside CK was 64.23 ± 0.41%. Additionally, it was observed that the addition of glycerol was beneficial to the synthesis of CK. When 20% glucose (C mol) in the YPD medium was replaced by the same C mol glycerol, CK production increased to 384.52 ± 15.23 mg/L, which was 45.68% higher than that in YPD medium, and the PPD conversion rate increased to 77.37 ± 3.37% as well. As we previously observed that ethanol is beneficial to the production of PPD, ethanol and glycerol were fed simultaneously in the 5-L bioreactor fed fermentation, and the CK levels reached 1.70 ± 0.16 g/L.

**Conclusions:**

In this study, we constructed an *S. cerevisiae* cell factory that efficiently produced ginsenoside CK. Glycerol effectively increased the glycosylation efficiency of PPD to ginsenoside CK, guiding higher carbon flow to the synthesis of ginsenosides and effectively improving CK production. CK production attained in a 5-L bioreactor was 1.7 g/L after simultaneous feeding of glycerol and ethanol.

## Background

Ginseng has been used in treatment of cardiovascular and cerebrovascular diseases, neurasthenia, and physical weakness, and ginsenosides are the primary ingredients in Ginseng that elicit these physiological activities [1]. Ginsenoside compound K (CK), the primary deglycosyl metabolite of ginsenosides produced under the action of intestinal flora and absorbed into the body, has been proven to elicit anticancer, anti-inflammatory, anti-allergic, antidiabetic, anti-angiogenic, anti-aging, neuroprotective, and liver protective properties among several notable biological characteristics [2]. Ginsenoside CK is a rare ginsenoside derived from ginseng saponins Rb1 and Rb2 by intestinal bacteria [3]. The method of synthesis of CK has garnered significant attention. Several studies have focused on the hydrolysis of major ginsenosides to active secondary ginsenosides using mild acid hydrolysis alkali treatment, microbial conversion, and enzymatic conversion [4–7].

Synthetic biological studies have seen persistent development in recent years, enabling microbial synthesis of compounds derived from animals and plants. For example, artemisinin is produced by *Saccharomyces cerevisiae* [8], phloroglucinol is produced by *Escherichia coli* [9], pinene is produced by *Corynebacterium glutamicum* [10] and so on. CK, a rare ginsenoside eliciting multiple biological activities, and can be synthesized using synthetic biological techniques as well. Zhou et al. synthesized 1.4 mg/L of CK using yeast [11]. Gwak et al. synthesized CK using tobacco as the host, and the concentration of CK achieved was 1.55–2.64 μg/g CDW [12]. Li et al. overexpressed the key genes of the mevalonate (MVA) pathway and the fusion of PPDS-ARR1 in *Yarrowia lipolytica*, and the CK production in 5-L bioreactor was 161.8 mg/L with fermentation optimization [13].

*S. cerevisiae* is a simple eukaryotic cell with a complete and mature eukaryotic expression system. Compared to the prokaryotic expression system, the complete organelle structure is beneficial to the further processing of proteins, thus ensuring the activity of enzymes. *S. cerevisiae* has a distinct genetic background and is biologically safe. *S. cerevisiae* is an important industrial host for the production of enzymes, drugs, and nutrients, as well as commercial chemicals and biofuels [14]. In CK synthesis, the glycosylation of PPD requires the participation of uridine diphosphate-glucose (UDPG). The supply of UDPG to yeast plays a significant role in glucoside biosynthesis [15]. Wang et al. overexpressed the gene *PGM2* and *UGP1* genes encoding phosphoglucomutase 2 and UTP-glucose-1-phosphate uridylyltransferase 1, respectively, in *S. cerevisiae* that are involved in the glucose synthesis system of uridine diphosphate. The conversion rate of scutellarein into its glucoside increased from 75 to 92% [16].

Glycerol has been established as a low cost waste product due to the global production of biodiesel [17]. Thus, new markets or novel applications of glycerol, such as conversion into valuable products, need to be explored [18]. Fermentation of glycerol by *Anaerobiospirillum* has been shown to produce succinic acid, while citric acid has been produced by *Yarrowia lipolytica*, 1,3-propanediol, acetic acid, and butyric acid by *Clostridium butyricum*, and 3-hydroxypropionic acid by *Klebsiella pneumoniae* [19].

Apart from its low cost, glycerol has several other advantages. Glycerol undergoes greater reduction than glucose, as its metabolism generates higher quantity of NAD(P)H [20]. Glycerol is metabolized through respiration manner in most microorganisms, including *S. cerevisiae*, and this implies that the use of glycerol does not exert the Crabtree effect in *S. cerevisiae* [21]. Glycerol can improve the stability of cell membrane proteins [22] and promote correct folding of nascent polypeptides [23]. Ginsenoside CK is a product of glycosylation of protopanaxadiol (PPD), and its production requires the participation of UDPG. Dutra et al. indicated that glycerol increases UDPG pyrophosphatase activity relative to glucose [24]. Wilson et al. observed that when *S. cerevisiae* is cultured in glycerol, the glycogen accumulated is more than that accumulated when it is cultured in glucose, and the precursor of glycogen synthesis is UDPG [25].

**Fig. 1:**
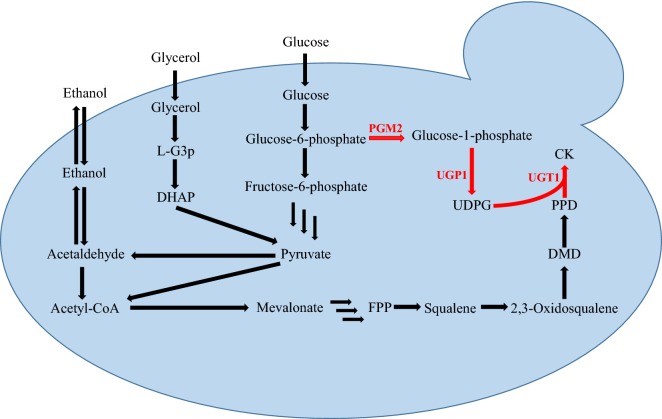
CK synthesis pathway in *Saccharomyces cerevisiae*. *PGM2* phosphoglucomutase 2, *UGP1* UTP-glucose-1-phosphate uridylyltransferase 1, *UGT1* UDP-glucose glucosyltransferase, *FPP* farnesyl diphosphate, *DMD* dammarenediol-II, *PPD* protopanaxadiol, *CK* compound K, *UDPG* uridine diphosphate-glucose

We constructed an *S. cerevisiae* strain WLT-MVA5 [26] that could produce PPD, and this strain is used as a parent strain for further engineering. The metabolic pathway is presented in Fig. 1. We overexpressed the genes associated with UDPG synthesis. Based on these advantages of glycerol, this study used glycerol as a partial carbon source and effectively increased the conversion efficiency of PPD to ginsenoside CK. We used a mixture of ethanol and glycerol to enable a CK production of 1.70 ± 0.16 g/L. To the best of our knowledge, this is the highest reported levels of CK production.

## Results and discussion

### Construction of strains producing CK

**Fig. 2:**
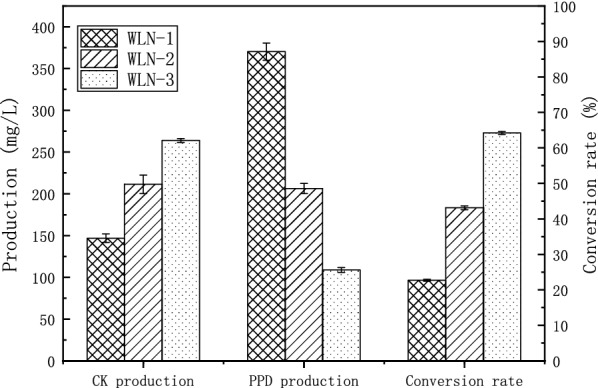
Comparison of fermentation by WLN-1, WLN-2, and WLN-3 strains. WLN-1, WLN-2, and WLN-3 were fermented in YPD medium for 96 h

In order to obtain a strain producing ginsenoside CK, the previously constructed PPD producing *S. cerevisiae* WLT-MVA5 [26] was used as a parent strain for further studies. First, we overexpressed the *UGT1* gene encoding glycosyltransferase that catalyzes the synthesis of CK from PPD. The *UGT1* gene expression cassette was inserted into the *ade2* site of the WLT-MVA5, and the strain WLN-1 was obtained. The CK production of WLN-1 at 96 h was 146.79 ± 5.11 mg/L, and the PPD production was 370.24 ± 10.23 mg/L (Fig. 2). The conversion rate from PPD to ginsenoside CK was 22.68 ± 0.29%. The conversion rate was low, and 77.32% of PPD remained unconverted. In order to promote the transformation of PPD, we tried increasing the UDPG supply. The gene *UGP1* encoding UTP-glucose-1-phosphate uridylyltransferase 1 which catalyzes the synthesis of UDPG from glucose-1-phosphate was overexpressed, and the strain WLN-2 was obtained. The CK production by WLN-2 reached 211.42 ± 11.01 mg/L at 96 h, the PPD production was 206.39 ± 6.32 mg/L, and the conversion rate was 43.11 ± 0.54%. In order to further increase the production of CK, we overexpressed the gene *PGM2* encoding phosphoglucomutase 2, which catalyzes the synthesis from glucose-6-phosphate to glucose-1-phosphate, and *PGK1p*-*PGM2*-*PGM2t*, *TDH3P*-*UGP1*-*UGP1t*, and *TEF1p*-*UGT1*-*ADH1t* expression cassettes were inserted into the single copy site *ade2* of *S. cerevisiae* WLT-MVA5 to obtain the strain WLN-3. The CK production of WLN-3 reached 263.94 ± 2.36 mg/L at 96 h, the PPD production was 108.75 ± 2.90 mg/L, and the conversion rate was 64.23 ± 0.4%. After overexpression of UDPG synthesis-related genes, the production of CK increased by 79.81%, and the conversion rate increased by 183.20%. As depicted in Fig. 2, CK production was limited by the supply of UDPG and increased by 79.81% after PGM2 and UGP1 overexpression compared to that in WLN-1.

### Effect exerted by glycerol as partial carbon source on CK production

**Fig. 3:**
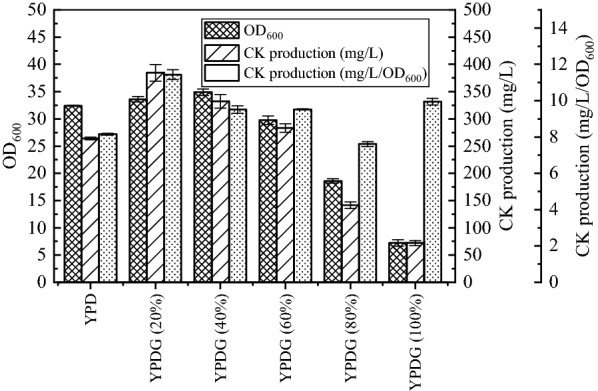
Effect of different glycerol to glucose ratios on CK production. YPDG (20%), YPDG (40%), YPDG (60%), YPDG (80%), YPDG (100%), respectively represent 20, 40, 60, 80, and 100% glucose in YPD replaced by glycerol of equimolar carbon quantity

Dutra et al. observed that glycerol increases UDPG pyrophosphatase activity relative to glucose [24]. We prepared a series of fermentation mediums using glucose and glycerol as carbon sources, that had the same molar concentration of carbon as YPD, and compared the effects of the medium with different glycerol to glucose ratios (see materials and methods) on CK production by WLN-3. The data is presented in Fig. 3. Based on Fig. 3, YPDG (20%) exhibited the highest CK production, YPDG (40%) exhibited the highest OD_600_. However, OD_600_ reduces with the increase in the proportion of glycerol (above 40%). YPDG (20%) is exhibits the highest increase in unit OD_600_, and apart from for YPDG (80%), the other media containing glycerol exhibit a higher increase in unit OD_600_ than YPD. Wilson et al. observed that when *S. cerevisiae* is cultured in glycerol, the levels of glycogen accumulated are greater than that when cultured in glucose, and the precursor of glycogen synthesis is UDPG [25]. Glycerol promotes CK production possibly because it increases the supply of UDPG or the activity of glycosyltransferases. Glycerol promotes cell growth possibly since (1) it is more reduced than glucose [20], (2) it improves the stability of cell membrane proteins [22], and (3) it promotes the correct folding of nascent polypeptides [23]. The OD_600_ of yeast cultured with pure glycerol as carbon source is 7.25, indicating that glycerol utilization is relatively low and inhibits yeast growth. Therefore, with reduction in glucose concentration and increase in glycerol concentration, OD_600_ increased at first and then decreased. With increase in glycerol concentration (above 20%), the yield of CK decreased significantly. When the glycerol concentration was above 40%, yeast growth was inhibited. When the glycerol concentration is 80%, the effect exerted by glycerol on CK production is stronger than the effect exerted on growth, and hence increase in unit OD_600_ is lower than that in the control; when glycerol concentration is 100%, the inhibitory effect of glycerol on cell growth exceeds the effect on CK production, and hence it is higher than that observed in the control. The presence of glycerol favors the synthesis of CK compared to presence of glucose in isolation, although the growth of the strain is dependent on glucose.

**Fig. 4:**
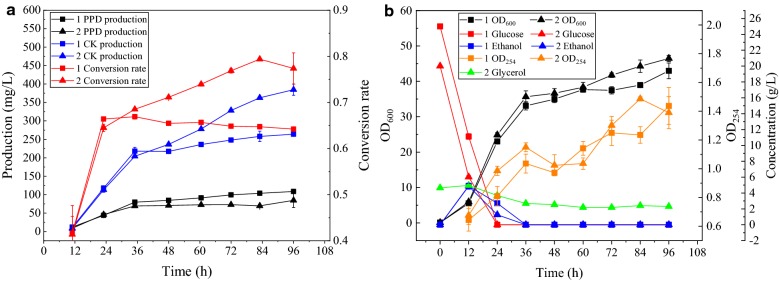
Comparison of fermentation process between YPD and 20% glycerol medium. **a** Alterations in production during fermentation. **b** Other parameters altered during fermentation. In the legend, 1 represents YPD, and 2 represents YPDG (20%) (20% of glucose in YPD is replaced by an equimolar carbon quantity of glycerol). OD_254_ represents the relative quantity of UDPG

From Fig. 3, it can be inferred that YPDG (20%) is most favorable for ginsenoside CK synthesis; hence, the fermentation in the medium with YPD and YPDG (20%) is monitored and OD_254_ is used to represent the relative concentration of UDPG (Fig. 4). Yeast utilizest glucose and produces ethanol simultaneously. After the depletion of glucose, the yeast begins to consume ethanol when YPD medium is used. However, ethanol and glycerol were co-consumed when the strain was cultivated in YPDG (20%) medium. At 36 h, ethanol was exhausted in both media, and glycerol consumption ceased in YPDG (20%). The production of PPD and CK in the two media had no significant difference before 36 h. However, the accumulation of UDPG in YPDG (20%) was higher than that in YPD, indicating that the presence of glycerol could promote UDPG accumulation. After 36 h, CK production of YPDG (20%) was higher than that of YPD, and the conversion rate of PPD to CK in YPDG (20%) was higher than that in YPD. Lastly, in YPD, the production of CK was 263.94 ± 2.36 mg/L, the conversion rate was 64.23 ± 0.41%, and the yield from glucose was 10.56 ± 0.09 mg/g. In YPDG (20%), the production of CK was 384.52 ± 15.23 mg/L, 45.68% higher than that in YPD, the conversion rate was 77.37 ± 3.37%, 13.14% higher than that in YPD, the yield from carbon (glucose and glycerol) was 15.60 ± 0.62 mg/g, 47.73% higher than that in YPD, and the total concentration of PPD and CK was 25.86% higher than that in YPD. The overall levels of UDPG in YPDG (20%) was marginally higher than that in YPD. The addition of glycerol promoted PPD glycosylation and induced greater carbon flow to ginsenosides synthesis.

### Fermentation in the 5-L bioreactor

**Fig. 5:**
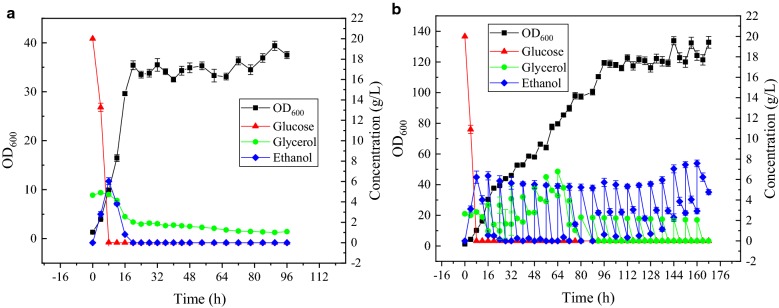
Fermentation process in a 5-L bioreactor. **a** Batch fermentation. **b** Fed-batch fermentation

For batch fermentation, YPDG (20%) medium was used as the fermentation medium, and 10% inoculum was used. The fermentation data is presented in Fig. 5a. At 8 h, glucose was depleted, while ethanol and glycerol consumption commenced; the ethanol concentration was lower than 1 g/L at 16 h, and the consumption rate of glycerol reduced. The production of CK at 96 h was 304.45 ± 16.23 mg/L, which was lower than that in the data from shake flask fermentation. On one hand, water volatilization in flask was more significant than that in the 5-L bioreactor, which inducing the concentration effect. On the other hand, the pool water solubility of CK made it adhere to the tank wall and stirring paddle during fermentation. In shake flask fermentation, the samples were collected by manual shaking. However, in the 5-L bioreactor, ginsenosides on the tank wall and stirring paddle could not be collected during the fermentation process, and the concentration of these ginsenosides was calculated using the initial loading volume. Therefore, both biomass and product titer in batch fermenter were lower than those in the flask.

For fed-batch fermentation, as shown in Fig. 5a, it was confirmed that the fermentation medium contained 2.65 g/L glycerol, 20 g/L glucose, 20 g/L peptone, and 10 g/L yeast extract. When pure glycerol is used as a carbon source, cells exhibit poor growth. We have previously demonstrated that ethanol is more favorable for the synthesis of PPD than glucose [27]. In YPDG (20%), ethanol and glycerol are simultaneously consumed after the glucose consumption, and the cells utilize glycerol negligibly after the ethanol is consumed. Since glycerol is beneficial to the formation of CK and ethanol is beneficial to the synthesis of PPD, we selected ethanol and glycerol for the feed. Based on the proportion and speed of consumption of ethanol and glycerol in batch fermentation, we commenced the feeding process at 16 h, adding 15 g of ethanol and 6.6 g of glycerol every 8 h; the fermentation data is presented in Fig. 5b. The final production levels achieved were 1.70 ± 0.16 g/L.

## Conclusion

We overexpressed the gene encoding the glycosyltransferase UGT1 and obtained a *Saccharomyces cerevisiae* strain that produced ginsenoside CK. Production was further increased by overexpression of UDPG supply-related genes. Our study indicated that glycerol can increase the conversion rate of PPD to CK. Fermentation conditions were optimized according to this feature. Lastly, the production levels in the 5-L bioreactor reached 1.7 g/L. Use of *S. cerevisiae* in synthesis of glycosylated natural products has been reported widely [16, 28, 29]. The findings from this study may be used to synthesize other glycosylated natural products using *S. cerevisiae*, and the cost of production can be reduced by using glycerin to synthesize natural products. Recent studies have indicated that *S. cerevisiae* can efficiently synthesize ethanol from glycerol [30] and in the future study, this strategy can be used to further increase CK production in *S. cerevisiae*.

## Materials and methods

### Strains and medium

The recently reported *S. cerevisiae* strain WLT-MVA5 was used as the parent strain [26]. The strain was stored at − 80 °C at 25% glycerol concentration. *S. cerevisiae* strains were cultured in YPD medium containing 25 g/L glucose, 20 g/L peptone, and 10 g/L yeast extract. *S. cerevisiae* strain transformants were cultured in SD medium lacking adenine, tryptophan, leucine, uracil, and histidine where appropriate [27].

### Strain construction

The *UGT1* (GenBank: AIE12479.1) gene, encoding UDP-glycosyltransferase, was synthesized and cloned into pUC57 plasmids by GENEWIZ (Suzhou, China), with codon optimization for *S. cerevisiae*. The genome of *S. cerevisiae* BY4741 was used for PCR amplification of *ADE2* genes. The genome of *S. cerevisiae* W303-1a was used for PCR amplification of *PGM2* and *UGP1* genes. *PGK1p*-*PGM2*-*PGM2t*, *TDH3p*-*UGP1*-*UGP1t*, and *TEF1p*-*UGT1*-*ADH1t* expression cassettes were constructed by fusion PCR. The expression cassettes were integrated into the *ade2* site of *S. cerevisiae* with selection marker ADE2.

### Flask fermentation

The initial fermentation medium consisted of YPD medium containing 25 g/L glucose, 20 g/L peptone, and 10 g/L yeast extract. A series of fermentation mediums using glucose and glycerol as carbon sources were prepared that, had the same molar concentration of carbon as YPD while they had different glucose to glycerol ratios. The carbon provided by glycerol accounted for 0, 20, 40, 60, 80, and 100% of the total carbon provided by glycerol and glucose, respectively. Accordingly, these media were abbreviated as YPD, YPDG (20%), YPDG (40%), YPDG (60%), YPDG (80%), YPDG (100%). The seeds for fermentation were cultured in YPD medium at 220 rpm, 30 °C for 18 h, and the conditions of flask fermentation were 220 rpm, fermentation at 30 °C for 4 days, and the initial inoculation OD was 0.1.

### Fermentation in the 5-L bioreactor

For batch fermentation in the 5-L bioreactor (Bailun, Shanghai, China), 200 mL of seed solution was cultured at 220 rpm, 30 °C for 18 h and inoculated into 2 L YPDG (20%) medium (20% of glucose in YPD replaced by equal molar glycerol) at a fermentation temperature of 30 °C and an air flow rate of 2 L/min. The dissolved oxygen (DO) is maintained at 40% or more by adjusting the stirring speed. The pH was maintained at 5.5 by automated addition of 2 M sulfuric acid and 5 M aqueous ammonia.

For 5-L fed-batch fermentation, the fermentation medium contained 2.65 g/L glycerol, 20 g/L glucose, 20 g/L peptone, and 10 g/L yeast extract. At 16 h after start of fermentation, 350 mL of feed solution (pH = 5.5) [26] and 25 mL of carbon source (glycerol 264 g/L, ethanol 600 g/L) were added. The carbon source was then added every 8 h. Other conditions are identical to those for a single batch fermentation.

### Metabolite extraction and analysis

Cell growth was determined by measuring the optical density at 600 nm (OD_600_) using a UV–VIS spectrophotometer. For the extraction and detection of PPD and CK, the fermentation broth was subjected to vortex agitation with n-butanol and 40–100 mesh SiO_2_. The mixture was then centrifuged, and the n-butanol phase was collected for HPLC analysis. HPLC analysis was conducted on Elite P230II high-pressure pump system equipped for UV detection at 203 nm. Chromatographic separation was performed using the Hypersil C18 column (4.6 mm × 250 mm, 5 μm; Elite Analytical Instruments Co., Ltd., Dalian, China). Acetonitrile–water (8:2, v/v) was used as the mobile phase. CK was determined by comparing the mass spectrogram with standards purchased from Meilun Biotechnology Co., Ltd (Dalian, China). Calculation of conversion rate from PPD to ginsenoside CK: PPD conversion rate = M_CK_/(M_CK_ + M_PPD_). M_CK_, molar concentration of CK; M_PPD_, molar concentration of PPD.

UDPG measurements refer to the study of Daran et al. [31]. The fermentation broth was centrifuged to discard the supernatant, shaken, and boiling ethanol was added thereto, maintained at the specific temperature for 5–10 min, and then freeze-dried. Three milliliter of 50 mM Tris (pH = 7.4) containing 1 mM EDTA was added to the residue. The absorbance at 254 nm was measured using a spectrophotometer.

## Supplementary information

**Additional file 1: Table S1.** Primers used for strains construction. **Figure S1.** LC/MS analysis of CK.

## Data Availability

All data supporting the conclusions of this article are included within the manuscript and Additional file 1.

## Abbreviations

PPD: Protopanaxadiol
CK: Compound K
UDPG: Uridine diphosphate-glucose
PGM2: Phosphoglucomutase 2
UGP1: UTP-glucose-1-phosphate uridylyltransferase 1
UGT1: UDP-glucose glucosyltransferase
FPP: Farnesyl diphosphate
DMD: Dammarenediol-II
