# Supplementary material for: Promotion of compound K production in Saccharomyces cerevisiae by glycerol
Source: Microb Cell Fact. 2020 Feb 19;19:41. doi: 10.1186/s12934-020-01306-3 (PMC7029525; doi:10.1186/s12934-020-01306-3)

**Additional file**

**Promotion of compound K production in Saccharomyces cerevisiae by glycerol**

**Microbial Cell Factories**

Weihua Nan^a^, Fanglong Zhao^a^, Chuanbo Zhang^a^, Haiyan Ju^a^, Wenyu Lu^a,b,c*^

^a^ School of Chemical Engineering and Technology, Tianjin University, Tianjin 300350, PR China;

^b^ Key Laboratory of System Bioengineering (Tianjin University), Ministry of Education, Tianjin, 300350, PR China;

^c^ SynBio Research Platform, Collaborative Innovation Center of Chemical Science and Engineering (Tianjin), Tianjin, 300350, PR China.

* Correspondence to: School of Chemical Engineering and Technology, Tianjin University, Tianjin 300072, PR China. Tel: +86-22-27892132, Fax: +86-22-27400973.

E-mail address: wenyulu@tju.edu.cn (W. Lu)

**Table S1**

Primers used for strains construction

| Primer name | Sequence (5' to 3') |
| --- | --- |
| ADE2R-f | ATGCTTGCGTCACTTCTCAAT |
| ADE2R-PGK1p-r | GTGCGTCTTGAGTTGAAGTCAGTCGAGCAAGAGCGCTTTAAA |
| PGK1p-ADE2R-f | TTTAAAGCGCTCTTGCTCGACTGACTTCAACTCAAGACGCAC |
| PGK1p-PGM2-r | GGAACCGTTTCAATTTGAAATGACATTGTTTTATATTTGTTGTAAAAAGTAGATAATTACTTCC |
| PGM2-PGK1p-f | GGAAGTAATTATCTACTTTTTACAACAAATATAAAACAATGTCATTTCAAATTGAAACGGTTCC |
| PGM2t-TDH3p-r | ACTCGAACTGAAAAAGCGTGTACCCAGTTGAACAATTCTGGT |
| PGM2t-f | AGGAACTGAAGAACCAACGGT |
| TDH3p-PGM2t-f | ACCAGAATTGTTCAACTGGGTACACGCTTTTTCAGTTCGAGT |
| TDH3p-UGP1-r | GGTGTGCTTCTTAGTGGACATTTTGTTTGTTTATGTGTGTTTATTCGAAAC |
| UGP1-TDH3p-f | GTTTCGAATAAACACACATAAACAAACAAAATGTCCACTAAGAAGCACACC |
| UGP1t-TEF1p-r | GGTGGTCGCTTTCTGTTGTATACGGGACAAATGTAACAAACGA |
| TEF1p-UGP1t-f | TCGTTTGTTACATTTGTCCCGTATACAACAGAAAGCGACCACC |
| TEF1p-GT-r | TGGCAAGAAAATCAATTCTGACTTCATTTTGTAATTAAAACTTAGATTAGATTGCTATGCTTT |
| GT-TEF1p-f | AAAGCATAGCAATCTAATCTAAGTTTTAATTACAAAATGAAGTCAGAATTGATTTTCTTGCCA |
| Gt-ADH1t-r | ACTTATTTAATAATAAAAATCATAAATCATAAGAAATTCGCTTACATAATTTCTTCGAATAATTTAGCCAATGA |
| ADH1t-Gt-f | TTATTCGAAGAAATTATGTAAGCGAATTTCTTATGATTTATGATTTTTATTATTAAATAAGT |
| ADH1t-ADE2L-r | GCTTCAAGCCGTTTAGCATTCTGACGATGAAGATAGAGCCCA |
| ADE2L-ADH1t-f | TGGGCTCTATCTTCATCGTCAGAATGCTAAACGGCTTGAAGC |
| ADE2L-r | CAATGGTATAATGTCCAGAGTTGTGA |
| ADE2R-TDH3p-r | ACTCGAACTGAAAAAGCGTGTGTCGAGCAAGAGCGCTTTAAA |
| TDH3p-ADE2R-f | TTTAAAGCGCTCTTGCTCGACACACGCTTTTTCAGTTCGAGT |
| ADE2R-TEF1p-r | GGTGGTCGCTTTCTGTTGTATGTCGAGCAAGAGCGCTTTAAA |
| TEF1p-ADE2R-f | TTTAAAGCGCTCTTGCTCGACATACAACAGAAAGCGACCACC |

**LC/MS operating conditions**

HPLC analysis was carried on an Elite P230II high-pressure pump system equipped with UV detection at 203 nm. Chromatographic separation was realized on Hypersil C18 column (4.6 mm×250 mm, 5μm; Elite Analytical Instruments Co., Ltd., Dalian, China). Acetonitrile-water (55:45, v/v) was used as mobile phase. MS operating conditions were as follows: source type, ESI; ion polarity, positive; all spectra were obtained over an m/z range of 50–1200; dry gas flow, 6.0 L/min; dry temperature, 180 ^o^C; nebulizer pressure, 0.8 bar; probe voltage +4.5 kV.

**Fig.S1**

**LC/MS analysis of CK**

**a** Liquid chromatography analysis results; **b** mass spectrometry results.


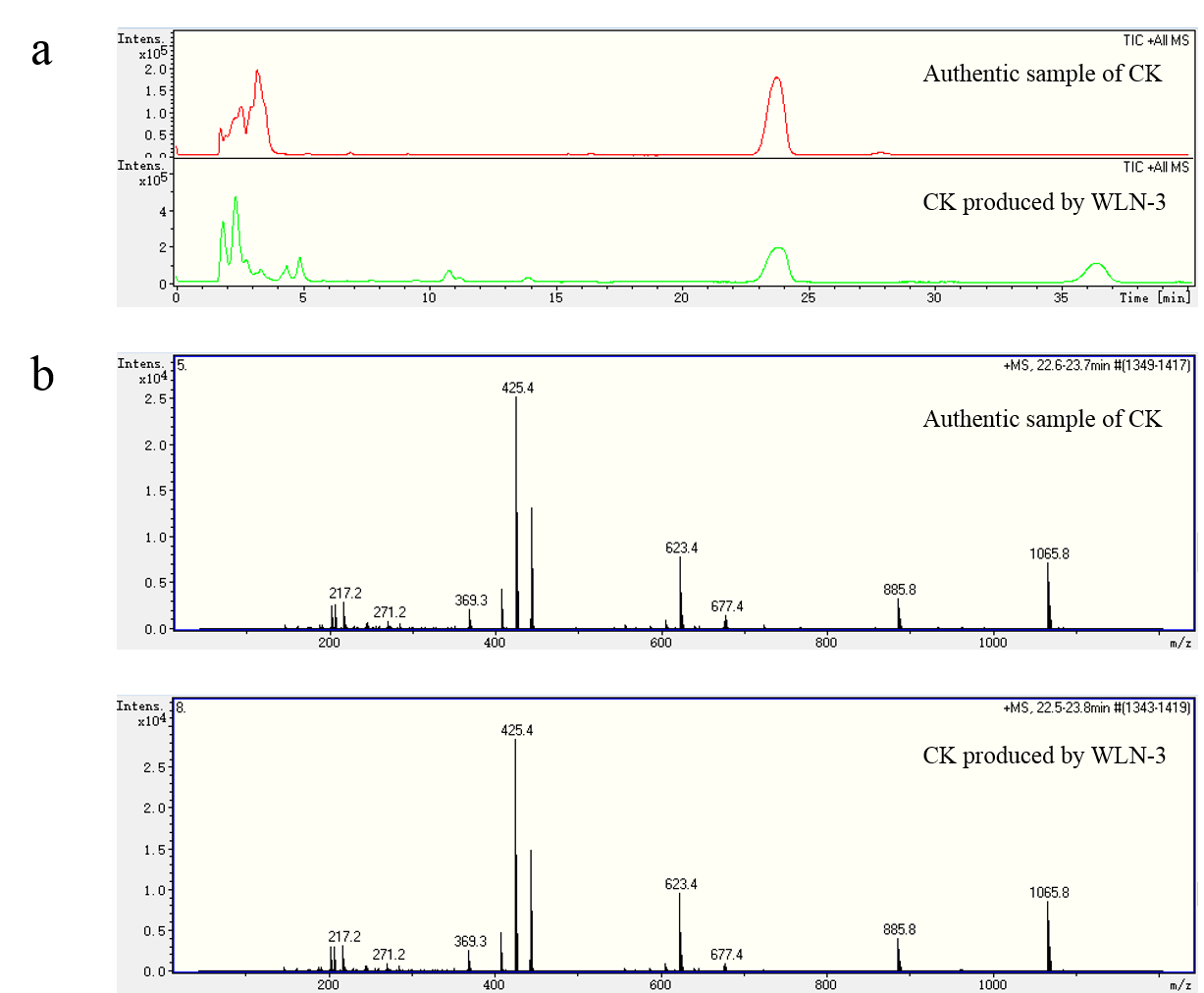

Supplement: Supplementary file 1 — Additional file 1: Table S1. Primers used for strains construction. Figure S1. LC/MS analysis of CK. [file 12934_2020_1306_MOESM1_ESM.doc]
